# Deep-learning analysis suggests a facial image signal associated with low muscle mass in a clinical cohort enriched for chronic liver disease: a proof-of-concept study

**DOI:** 10.3389/fcell.2026.1964880

**Published:** 2026-10-12

**Authors:** Thomas Fuchs, Akhila Abdulnazar, Lavra Rebol, Peter Fickert, Thomas Gremsl, Vanessa Stadlbauer

**Affiliations:** 1 Department of Gastroenterology and Hepatology, Division of Internal Medicine, Medical University of Graz, Graz, Austria; 2 CBmed GmbH - Center for Biomarker Research in Medicine, Graz, Austria; 3 Department of Ethics and Social Studies, Faculty of Catholic Theology, University of Graz, Graz, Austria

**Keywords:** artificial intelligence (AI), face recognition, liver, muscle, sarcopaenia

## Abstract

**Background:**

Sarcopenia is a clinically relevant complication of chronic liver disease. Because changes in muscle mass, adiposity, and nutritional status may affect facial morphology, this exploratory study examined whether standardized frontal facial photographs contain an algorithmically detectable signal associated with low measured muscle mass.

**Methods:**

Prospectively collected clinical data and facial photographs from 255 participants were retrospectively analyzed using the 2010 European Working Group on Sarcopenia in Older People (EWGSOP1) framework. The binary target was low versus normal measured muscle mass. Stage 1 developed ImageNet-pretrained VGG16, MobileNetV2, and DenseNet121 models in 204 participants and evaluated them in a 51-participant internal hold-out set. Stage 2 independently redeveloped the task using four region-specific EfficientNet-B0 and DenseNet121 components integrated into a single decision pipeline and evaluated it separately within the same source cohort. A clinical-variable-only comparator, human image assessment, Grad-CAM, and controlled perturbation analyses were also performed.

**Results:**

Overall, 173 participants had low and 82 had normal muscle mass. Stage-1 balanced accuracy was 0.71 for VGG16, 0.70 for DenseNet121, and 0.63 for MobileNetV2 The integrated stage-2 system showed high sensitivity (94.3%) but limited specificity (50.0%), resulting in a balanced accuracy of 0.72. Human image assessment showed low interrater agreement. Grad-CAM and perturbation analyses indicated contributions from facial regions but also substantial dependence on peripheral and non-face image content. The clinical-variable comparator outperformed the stage-1 facial models.

**Conclusion:**

Standardized facial photographs contained a image-derived information associated with low measured muscle mass across two independently implemented modelling stages. The integrated stage-2 pipeline achieved high sensitivity, supporting further investigation of facial image analysis as a low-burden pre-screening approach. However, limited specificity, potential clinical and diagnostic-pathway confounding, dependence on non-face image content, and the absence of external validation indicate that these findings remain exploratory. Prospective external validation and assessment of incremental value beyond routine clinical variables are required before clinical implementation.

## 1 Introduction

Sarcopenia is a progressive skeletal muscle disorder characterized by loss of muscle strength, muscle quantity or quality, and physical performance and is associated with frailty, falls, functional decline, complications, and mortality (Sayer and Cruz-Jentoft, 2022; Cruz-Jentoft et al., 2010; Cruz-Jentoft et al., 2019). It is particularly relevant in chronic liver disease, especially cirrhosis, where it commonly coexists with malnutrition and frailty and may adversely affect clinical outcomes and patient management (Lai et al., 2021; Tantai et al., 2022). Early detection is therefore important in clinical risk populations, as it may enable improved risk stratification and targeted diagnostic, nutritional, physical, and disease-specific interventions (Lai et al., 2021).

Established sarcopenia case-finding and diagnostic pathways combine clinically accessible measures such as handgrip strength, chair-rise performance, and gait speed with body-composition assessment using dual-energy X-ray absorptiometry, computed tomography, magnetic resonance imaging, bioelectrical impedance analysis, or other validated methods (Cruz-Jentoft et al., 2010; Cruz-Jentoft et al., 2019; Lai et al., 2021; Giovannini et al., 2021). Facial image analysis should therefore not be considered a replacement for established case-finding or diagnostic procedures. Its potential future role would be as a complementary, contactless pre-screening approach in settings in which conventional assessment is not immediately available. Whether such an approach can provide sufficient accuracy, scalability, cost-effectiveness, and clinical benefit remains unknown (Qiang et al., 2022).

Changes in muscle mass, adiposity, and nutritional status may affect craniofacial soft tissues, and facial image analysis has been investigated for genetic, endocrine, metabolic, and nutritional phenotypes (Qiang et al., 2022; Wu et al., 2024; Chen et al., 2023; Steindl et al., 2020; Wang et al., 2024). In addition, temporal and periorbital morphology and temporalis muscle thickness have been associated with systemic muscle or fat loss, while deep-learning analysis of three-dimensional facial information has been investigated for malnutrition classification (Chen et al., 2023; Steindl et al., 2020; Wang et al., 2024). These observations provide biological plausibility for facial photograph–based sarcopenia risk stratification. However, they do not establish that two-dimensional frontal facial photographs contain a strong or sarcopenia-specific signal. Age, body mass index, malnutrition, cachexia, underlying disease, and image-acquisition conditions may produce correlated visual patterns that are not specific to sarcopenia (Wu et al., 2024; Chen et al., 2023; Steindl et al., 2020; Zoltie et al., 2022).

Therefore, this exploratory study investigated whether standardized frontal facial photographs contain an algorithmically detectable signal associated with low measured muscle mass under the historical EWGSOP1-based classification in a mixed single-center clinical cohort enriched for chronic liver disease (Cruz-Jentoft et al., 2010). The implications of the subsequently revised 2019 criteria were considered when interpreting the findings (Cruz-Jentoft et al., 2019). In addition, human interrater agreement was assessed to determine whether unassisted visual classification of the same facial photographs was reproducible.

## 2 Materials and methods

### 2.1 Study design and study population

This exploratory diagnostic modeling study was a retrospective secondary analysis of prospectively collected clinical data and standardized facial photographs. Data were obtained from studies conducted at the Medical University of Graz, including the registered prospective controlled single-center study “Microbiome and Sarcopenia in Patients With Liver Cirrhosis” (ClinicalTrials.gov identifier: NCT03080129). The parent study began on April 11, 2017, and acquisition of the facial photographs used in the present analysis began in December 2019. The analytic dataset was frozen in January 2025. Both modelling stages used this same frozen source cohort, and no additional participants were introduced between stages. The study framework was subsequently extended through the prospective biobanking protocol “Sarcopenia in Cirrhosis and Chronic Inflammatory Bowel Disease: An Austrian Biobanking Study” which explicitly included the development of a neural network to distinguish facial photographs of participants with and without sarcopenia as a secondary objective.

Adults older than 18 years with a clinical, radiologic, or histologic diagnosis of liver cirrhosis or chronic liver disease, or a histologically confirmed diagnosis of inflammatory bowel disease, were eligible under the amended study protocol. Additional inclusion criteria for the present analysis were written informed consent and availability of an eligible body-composition assessment by computed tomography, magnetic resonance imaging, or dual-energy X-ray absorptiometry within 2 months before or after the study visit. Exclusion criteria included hepatic encephalopathy above grade 2 or another cognitive disorder precluding informed consent, hepatocellular carcinoma classified as Barcelona Clinic Liver Cancer stage C or D, and any other condition considered by the investigator to interfere with study participation.

For the present analysis, participants were required to have an available standardized frontal facial photograph and sufficient clinical data to derive the study reference classification. The analysis cohort comprised participants with liver cirrhosis, participants with inflammatory bowel disease, and control participants. It therefore represents a mixed single-center clinical cohort enriched for liver cirrhosis rather than a population-based sample or an exclusively hepatologic cohort. The final sample size and the distributions of disease groups and reference classifications are reported in the Results. The frozen analytic master dataset comprised 255 participants, all of whom had available facial photographs and a complete EWGSOP1-derived reference classification.

Participants were identified through the clinical services of the Department of Gastroenterology and Hepatology and were approached during routine outpatient visits or inpatient stays. The amended protocol also included participants with inflammatory bowel disease.

The original study was approved by the Ethics Committee of the Medical University of Graz under approval number 29–280 ex 16/17. Approval number 34–294 ex 21/22 constituted an amendment to the original study protocol. All participants provided written informed consent before study procedures, and the study was conducted in accordance with the Declaration of Helsinki.

### 2.2 Reference standard for sarcopenia

The study reference classification was based on the 2010 European Working Group on Sarcopenia in Older People framework (Cruz-Jentoft et al., 2010; Traub et al., 2020). The EWGSOP1 classification comprised four categories: no sarcopenia, presarcopenia, sarcopenia, and severe sarcopenia. Participants classified as having no sarcopenia had normal measured muscle mass, whereas all three remaining stages required low muscle mass (Cruz-Jentoft et al., 2010; Traub et al., 2020).

For the present binary image-classification analysis, participants with normal measured muscle mass were assigned to the negative class and participants with low measured muscle mass to the positive class. Under the historical EWGSOP1 classification, this corresponded exactly to combining presarcopenia, sarcopenia, and severe sarcopenia into the positive class while assigning participants without sarcopenia to the negative class. Accordingly, the binary machine-learning target was low versus normal measured muscle mass and should not be interpreted as classification of strict or contemporary sarcopenia.

Muscle mass was assessed using the body-composition modality available in the clinical source dataset, including computed tomography, magnetic resonance imaging, or dual-energy X-ray absorptiometry. For computed tomography and magnetic resonance imaging, skeletal muscle area was quantified on an axial image at the level of the third lumbar vertebra using Slice-O-Matic software (Tomovision, version 6 rev 13). The segmented muscles included the psoas, paraspinal, transversus abdominis, internal and external oblique, rectus abdominis, and quadratus lumborum muscles. Skeletal muscle area was normalized to height squared and expressed as the lumbar skeletal muscle index. Low muscle mass was defined as a lumbar skeletal muscle index of ≤52.4 cm^2^/m^2^ in men and ≤38.5 cm^2^/m^2^ in women (Traub et al., 2020; Montano-Loza et al., 2012). Computed tomography and magnetic resonance imaging assessments were performed by a single operator blinded to participant group.

For participants assessed using dual-energy X-ray absorptiometry, appendicular skeletal muscle mass was normalized to height squared. Low muscle mass was defined using the sex-specific Rosetta-study cut-offs reported in the EWGSOP1 consensus, corresponding to an appendicular skeletal muscle mass index below 7.26 kg/m^2^ in men and below 5.50 kg/m^2^ in women (Cruz-Jentoft et al., 2010).

Muscle strength was assessed using dominant-hand grip strength measured with a Jamar hydraulic hand dynamometer. Low muscle strength was defined using the sex- and body mass index specific cutoff values reported within the 2010 European Working Group on Sarcopenia in Older People framework. For men, the cutoff values were ≤29 kg for a body mass index ≤24 kg/m^2^, ≤30 kg for a body mass index of 24.1–28.0 kg/m^2^, and ≤32 kg for a body mass index >28 kg/m^2^. For women, the cutoff values were ≤17 kg for a body mass index ≤23 kg/m^2^, ≤17.3 kg for a body mass index of 23.1–26.0 kg/m^2^, ≤18 kg for a body mass index of 26.1–29.0 kg/m^2^, and ≤21 kg for a body mass index >29 kg/m^2^ (Lai et al., 2021). The single dominant-hand grip-strength value available in the clinical source dataset was used for classification.

EWGSOP1 was retained as the reference framework because it was prospectively specified in the parent study and formed the target on which the image-classification task was originally defined. This choice was particularly relevant to the cirrhosis-enriched study population because previous work from the study group demonstrated materially different classification under the 2010 and 2019 EWGSOP frameworks (Traub et al., 2020). Although an EWGSOP2-derived classification is available in the source dataset, retrospective substitution of EWGSOP2 as an alternative model target was considered a distinct diagnostic research question rather than a technical relabelling of the present study.

### 2.3 Facial photograph acquisition and preprocessing

Facial photographs were acquired by trained study personnel during the baseline study visit using a smartphone and the Pics4Health study application. Because facial photography was performed during the study visit, the protocol-defined interval between facial image acquisition and the CT-, MRI-, or DXA-based body-composition assessment was at most 2 months in either direction. Participant-level timing distributions were not retrospectively derived beyond this protocol-specified interval.

The photographs were therefore standardized clinical photographs and not participant-captured selfies. According to the study protocol, the application transferred the images together with a pseudonymous study code to secure Microsoft Azure cloud storage, and no images were stored permanently on the acquisition device. The study code and directly identifying participant information were managed separately.

Photographs were obtained under standardized conditions with the participant positioned frontally. Participants were instructed to maintain a neutral facial expression, and a uniform background was used.

Faces were automatically detected and localized using RetinaFace (Deng et al., 2020). RetinaFace generated a facial bounding box, after which an additional margin was included around the detected facial region to retain sufficient surrounding facial context. The resulting crop was resized to 224 × 224 pixels before being supplied to the deep-learning pipeline. RetinaFace was used for face detection and localization; no identity recognition or matching was performed.

### 2.4 Human image assessment

To assess the reproducibility of unassisted visual classification, an annotation study was conducted with three rater groups: experienced clinicians with more than 6 years of professional experience, junior clinicians with less than 6 years of professional experience, and medical students without professional clinical experience. Raters were recruited by email and reviewed the facial photographs through a secure Amazon SageMaker Ground Truth interface (Amazon Web Services, Seattle, WA, United States).

Raters were blinded to participant characteristics, clinical measurements, disease group, and the study reference classification. For each photograph, they were required to select either “sarcopenic” or “non-sarcopenic.” Skipping an image was not permitted, and submitted decisions were final. Because no clinical information was provided, the task represented visual classification based on facial appearance alone. Raters received the written instructions accompanying the departmental annotation request but did not undergo a formal training set, calibration session, or feedback-based training before classification.

For the model-comparison analysis reported here, the analysis was restricted to the 51 photographs constituting the internal hold-out set. All 14 raters classified each of these 51 photographs, yielding 714 complete ratings without missing observations; no missing-rating imputation was therefore required. The original departmental response comprised three experienced clinicians, four junior clinicians, and seven medical students.

Repeated assessments of the same images were not collected. Consequently, intra-rater or test-retest reliability could not be evaluated. Two photographs resulted in an exact 7:7 pooled vote.

### 2.5 Stage 1: initial model development and internal hold-out evaluation

Stage 1 evaluated three ImageNet-pretrained convolutional neural-network architectures: VGG16, MobileNetV2, and DenseNet121 (Simonyan and Zisserman, 2014; Sandler et al., 2018; Huang et al., 2017). Participants were separated at participant level into a 204-participant development set and an internal 51-participant hold-out set, with one photograph per participant. Model development, hyperparameter selection, and threshold selection were restricted to the development dataset, and the hold-out set was used for final evaluation. The models were initialized using weights pretrained on ImageNet-1K and adapted to the present classification task by transfer learning (Russakovsky et al., 2015). The convolutional feature-extraction layers were frozen, and each architecture was extended with a task-specific classification head comprising a fully connected layer, rectified linear unit activation, dropout, and a final linear layer producing one logit.

The binary target distinguished participants with low measured muscle mass from participants with normal measured muscle mass. Under the historical EWGSOP1 classification, the low-muscle-mass class comprised presarcopenia, sarcopenia, and severe sarcopenia, whereas the normal-muscle-mass class corresponded to participants in the EWGSOP1 “no sarcopenia” category.

The hold-out set was selected from the subset with human annotations. Each participant contributed one photograph, and no participant was included in more than one data partition.

All model inputs consisted of 224 × 224 RGB images. Pixel channels were normalized using the ImageNet parameters mean = [0.485, 0.456, 0.406] and standard deviation = [0.229, 0.224, 0.225]. Input tensors were arranged in channels-first format as [batch, channel, height, width].

Training images were augmented using random resized cropping, horizontal flipping, rotation, and color jitter. Augmentation was applied only to the training partitions. Validation and hold-out images were resized and normalized without random augmentation.

Hyperparameters were optimized by grid search using repeated stratified five-fold cross-validation within the development set. Models were trained using binary cross-entropy loss with Adam optimization and inverse class-frequency weighting. A ReduceLROnPlateau learning-rate scheduler and early stopping were used to reduce overfitting. Exact scheduler parameters, early-stopping settings, random seeds, software-library versions, augmentation parameter ranges, and the total number of candidate configurations could not be recovered with sufficient certainty and were therefore not retrospectively inferred.

Hyperparameter selection for the first-generation models was performed within the 204-participant development dataset. Archived documentation from the original model-development analysis permitted recovery of the final selected configurations. For VGG16, the selected learning rate was 0.0001, dropout 0.3, fully connected-layer size 128 units, weight decay 0.01, and decision threshold 0.55. For MobileNetV2, the corresponding values were 0.001, 0.5, 256 units, 0.001, and 0.40. For DenseNet121, they were 0.0001, 0.5, 128 units, 0.01, and 0.50. The best validation balanced accuracies recorded during model selection were 0.816, 0.810, and 0.778, respectively. Model and threshold selection were completed before evaluation in the 51-participant hold-out set.

For each architecture, the decision threshold was selected exclusively within the cross-validation procedure to maximize balanced accuracy. After completion of model and threshold selection, the final configuration was evaluated in the internal hold-out set.

### 2.6 Stage 2: independent redevelopment and internal validation

Stage 2 was designed to test whether the discrimination observed in stage 1 could be reproduced after independent redevelopment of the computational pipeline. The same binary target and frozen source cohort were retained, but preprocessing, region-of-interest construction, model implementation, and model architecture were independently redeveloped by a second investigator. Stage 2 therefore served as an internal replication and validation stage within the same source cohort and should not be interpreted as external validation.

The stage-2 pipeline comprised four region-specific convolutional neural-network components. An EfficientNet-B0 model analyzed a full-face-no-neck representation, a second EfficientNet-B0 model analyzed a masked full-face-no-neck representation, a DenseNet121 model analyzed an aligned midface representation, and a second DenseNet121 model analyzed an aligned eye-cheek representation. All model inputs were represented as 224 × 224 RGB images and normalized using ImageNet mean and standard-deviation parameters.

The stage-2 decision framework combined the component probabilities using prespecified decision rules. A sensitive screening score was calculated as the mean of the full-face-no-neck and eye-cheek scores and was considered positive at ≥0.44. A risk score was calculated as the mean of the full-face-no-neck and midface scores and was considered positive at ≥0.54. A high-specificity score was calculated as the mean of the masked full-face-no-neck and eye-cheek scores and was considered positive at ≥0.60 when the risk criterion was simultaneously fulfilled. For the binary internal validation reported here, the system was considered positive when either the sensitive or risk criterion was met. The complete composite decision rules and their respective thresholds are summarized in Supplementary Table S6.

Stage-2 internal evaluation was performed in a 51-participant test partition common to all four model components. Performance was summarized using the confusion matrix, sensitivity, specificity, positive predictive value, negative predictive value, accuracy, and balanced accuracy. Because both modelling stages originated from the same clinical source cohort, stage 2 was interpreted as an internal replication and validation of the image-associated discrimination across developers, preprocessing strategies, and model architectures, but not as evidence of external generalizability.

The retained computational environment used for the stage-2 analysis and model audit comprised Python 3.12.3 running under Windows Subsystem for Linux 2 (WSL2 2.3.26.0) with Ubuntu 24.04.4 LTS. Core framework versions were PyTorch 2.12.1, torchvision 0.27.1, and scikit-learn 1.9.0; complete environment details are provided in Supplementary Table S5.

### 2.7 Clinical-variable comparator

To contextualize facial-model performance against routinely available clinical and diagnostic-pathway information, an exploratory clinical-variable-only comparator was developed using the same participant-level development/hold-out separation as the first-generation facial models. The binary outcome was low versus normal measured muscle mass. Predictors comprised age, sex, body mass index, disease group, and reference-standard modality. Implausible age and body mass index values were treated as missing according to the predefined plausibility criteria. Missing continuous values were imputed using development-set medians, continuous predictors were standardized, and categorical predictors were one-hot encoded. All preprocessing steps were fitted exclusively within the 204-participant development set.

The comparator was implemented as an L2-regularized logistic-regression model. The regularization parameter C was selected by stratified five-fold cross-validation within the 204-participant development set from candidate values of 0.01, 0.03, 0.1, 0.3, 1, 3, and 10. The final selected value was C = 0.3. A probability threshold of 0.66 was selected from development-set out-of-fold predictions to maximize balanced accuracy. The final model was then refitted using the complete development set and evaluated once in the 51-participant stage-1 internal hold-out set.

Because reference-standard modality was itself strongly associated with the outcome, an additional sensitivity analysis repeated the clinical comparator without imaging modality, using age, sex, body mass index, and disease group only.

The clinical-variable comparator was implemented in the same Python/WSL2 environment using scikit-learn 1.9.0, with NumPy 2.5.0 and pandas 3.0.3 for data processing.

### 2.8 Post-hoc interpretability and perturbation audit of stage 2

Interpretability analysis was performed on the stage-2 pipeline. The following analyses are specific to the stage-2 networks and were not used to retrospectively explain individual predictions of the stage-1 model.

Grad-CAM (Selvaraju et al., 2020) was calculated for all four second-generation components across the complete 51-participant internal test set. True-positive, true-negative, false-positive, and false-negative categories were assigned according to the final system prediction rather than to the output of individual component networks. Individual Grad-CAM maps were generated for every participant, and aggregate maps were constructed separately for the four system-level outcome categories. Representative examples were selected algorithmically rather than on the basis of visual appearance.

For the full-face-no-neck component, normalized Grad-CAM activation was additionally quantified within prespecified geometric image-space proxy regions representing the approximate facial envelope, background, outer 10% image border, upper and lower non-face areas, and temporal, periorbital, and cheek regions. These masks were used as reproducible image-space audit regions and were not intended to represent exact anatomical segmentation.

Controlled sensitivity analyses were subsequently performed by individually masking the background, outer image border, upper or lower non-face regions, temporal, periorbital, or cheek proxy regions, converting the input to grayscale, or applying a 10% tighter crop. For each perturbation, the other three component scores were held unchanged and the complete system decision was recalculated. Because masking and crop modification may themselves produce out-of-distribution inputs, these analyses were interpreted as model-sensitivity tests rather than causal explanations.

### 2.9 Statistical analysis

Overall interrater reliability across the 14 raters was quantified using Krippendorff’s alpha for nominal data (Marzi et al., 2024). Uncertainty was estimated by nonparametric bootstrap resampling of photographs with 10,000 repetitions. Individual-rater balanced accuracies were additionally calculated against the binary low-muscle-mass reference classification and summarized using the median and range. A bootstrap confidence interval was calculated for the median individual-rater balanced accuracy.

The primary performance measure for the deep-learning models was balanced accuracy, defined as the arithmetic mean of sensitivity and specificity. Sensitivity was defined as the proportion of participants with low measured muscle mass who were classified as positive. Specificity was defined as the proportion of participants with normal measured muscle mass who were classified as negative. Secondary measures included overall accuracy, precision, recall, F1 score, and confusion-matrix counts.

The study was not designed or powered for a formal statistical test of superiority between human raters and the deep-learning models. Human judgments and model predictions were therefore compared descriptively using their respective point estimates and uncertainty measures.

Clinical, demographic, and laboratory analyses were performed using IBM SPSS Statistics, version 30 (IBM Corp., Armonk, NY, United States). Continuous variables were summarized using mean, standard deviation, median, minimum, and maximum. Categorical variables were summarized using counts and percentages.

Comparisons between the low- and normal-muscle-mass groups in the overall cohort were performed using the Mann-Whitney U test for continuous variables and the chi-square test or Fisher’s exact test for categorical variables, as appropriate.

Before clinical analyses, predefined plausibility checks were applied. Values were set to missing if age was <18 or >100 years, height was <120 or >220 cm, body mass index was <10 or >60 kg/m^2^, serum sodium was <120 or >160 mmol/L, or mid-arm muscle circumference was <100 or >350 mm. Plausibility-cleaned variables were used for subsequent descriptive and exploratory analyses.

Variables considered relevant to interpretation of the facial classification task included age, body mass index, sex, disease group, reference-standard imaging modality, Child–Pugh score, and Model for End-Stage Liver Disease score. Differences between reference-label groups were interpreted as cohort and label imbalances. They were not interpreted as evidence that the image models had used the corresponding clinical variables or facial correlates as predictive features.

Laboratory variables were analyzed on a complete case basis. Because availability differed between laboratory parameters and numerous unadjusted comparisons were performed, these analyses were regarded as exploratory contextual analyses and detailed results were assigned to the Supplementary Material.

Hold-out-set distributions were reported primarily descriptively. Any *post hoc* inferential comparisons performed after final model evaluation were explicitly treated as exploratory and were not considered part of model development or validation.

Predictive values were reported descriptively for the internal evaluation samples and were not extrapolated to an assumed intended-use prevalence.

## 3 Results

### 3.1 Study population

By construction of the EWGSOP1 classification, all 173 participants assigned to the positive binary class had low measured muscle mass, whereas all 82 participants assigned to the negative class had normal measured muscle mass. Thus, the binary machine-learning outcome was equivalent to low versus normal measured muscle mass. Of the 173 participants with low muscle mass, 97 (56.1%) had presarcopenia, 58 (33.5%) sarcopenia, and 18 (10.4%) severe sarcopenia.

After exclusion of one implausible age value according to the predefined plausibility criteria, the mean age was 58.4 years among 254 participants. Overall, 96 participants were female and 159 were male. The cohort comprised 141 participants with liver cirrhosis, 44 with inflammatory bowel disease, and 70 control participants.

The internal hold-out set included 51 participants, of whom 36 were assigned to the low-muscle-mass class and 15 to the negative class. It comprised 35 participants with liver cirrhosis and 16 control participants; no participants with inflammatory bowel disease were included. The positive class was therefore overrepresented in both the overall cohort and the hold-out set, and the disease-group composition of the hold-out set differed from that of the complete cohort. Participant characteristics are presented in Table 1.

**TABLE 1:** Baseline characteristics of the overall cohort and stage-1 internal hold-out set according to binary low-muscle-mass classification.

​	​	Total					Test set				
Characteristic	Category	Low-muscle-mass class		Normal-muscle-mass class		Total	Low-muscle-mass class		Normal-muscle-mass class		Total
​	Total	173	​	82	​	255	36	​	15	​	51
Sex	Female	60	34.7%	36	43.9%	96	10	27.8%	6	40.0%	16
	Male	113	65.3%	46	56.1%	159	26	72.2%	9	60.0%	35
Disease	Control	57	32.9%	13	15.9%	70	15	41.7%	1	6.7%	16
	Liver cirrhosis	80	46.2%	61	74.4%	141	21	58.3%	14	93.3%	35
	IBD	36	20.8%	8	9.8%	44	0	0.0%	0	0.0%	0
Age	Mean [years]	57.6 (55.1.-60.1); n = 172		60.0 (57.1–62.9); n = 82		58.4 (56.4–60.3); n = 254	55.4 (50.2–60.6)		63.7 (58.8–68.7)		57.9 (53.9–61.9)
Age group	<50 years	51	29.7%	16	19.5%	67	12	33.3%	1	6.7%	13
	50–64 years	53	30.8%	31	37.8%	84	12	33.3%	6	40.0%	18
	65–74 years	39	22.7%	25	30.5%	64	8	22.2%	7	46.7%	15
	≥75 years	29	16.9%	10	12.2%	39	4	11.1%	1	6.7%	5
BMI	Underweight (<18.5 kg/m^2^)	6	3.5%	0	0.0%	6	1	2.4%	0	0.0%	1
	Normal weight (18.5- <25 kg/m^2^)	81	47.1%	23	28.0%	104	18	51.4%	2	13.3%	20
	Overweight (25- <30 kg/m^2^)	72	41.9%	30	36.6%	102	13	37.1%	8	53.3%	21
	Obesity (>30 kg/m^2^)	13	7.6%	29	35.4%	42	3	8.6%	5	33.3%	8
Imaging method	CT	46	26.6%	23	28.0%	69	8	22.2%	5	33.3%	13
	MRI	121	69.9%	21	25.6%	142	27	75.0%	2	13.3%	29
	DXA	6	3.5%	38	46.3%	44	1	2.8%	8	53.3%	9

Values are presented as n (%) for categorical variables and as mean (95% confidence interval) for age. Percentages are column percentages and were calculated using the number of non-missing observations within each respective column. One implausible age value was treated as missing according to the predefined plausibility criteria, resulting in age data for 254 participants in the overall cohort (172 with low muscle mass and 82 with normal muscle mass). One implausible body mass index value was treated as missing, resulting in BMI, data for 254 participants in the overall cohort (172 with low muscle mass and 82 with normal muscle mass) and 50 participants in the stage-1, internal hold-out set (35 with low muscle mass and 15 with normal muscle mass). Under the historical EWGSOP1 classification, the normal-muscle-mass class corresponded to participants classified as having no sarcopenia, whereas the low-muscle-mass class comprised participants with presarcopenia, sarcopenia, or severe sarcopenia. Muscle mass was assessed using computed tomography, magnetic resonance imaging, or dual-energy X-ray absorptiometry according to the available clinical assessment. IBD, inflammatory bowel disease; BMI, body mass index; CT, computed tomography; MRI, magnetic resonance imaging; DXA, dual-energy X-ray absorptiometry. Table 2. Interrater agreement and majority-vote performance of the human-rater groups in the internal hold-out set.

Handgrip strength, gait speed, and chair-rise performance are summarized in Supplementary Tables S1–S3. Dominant handgrip strength was lower in the positive class, particularly among men, whereas gait speed and chair-rise performance showed substantial overlap between the binary classes. This overlap was expected because the positive class included participants with presarcopenia, defined by low muscle mass without necessarily reduced muscle strength or impaired physical performance.

### 3.2 Human image assessment

All 14 raters classified each of the 51 photographs in the internal hold-out set, yielding 714 complete image classifications without missing observations. Overall interrater agreement was low, with a Krippendorff’s alpha of 0.28 (95% bootstrap CI, 0.17–0.39). Individual-rater balanced accuracy had a median of 0.49 (range, 0.41–0.58; 95% bootstrap CI for the median, 0.40–0.57). Two photographs resulted in an exact 7:7 pooled vote.

These results demonstrate low reproducibility of unaided image-only classification under the experimental conditions and should not be interpreted as a measure of clinical diagnostic judgment or as validation of the neural-network models. Human-rater results are summarized in Table 2.

**TABLE 2:** Inter-annotation agreement measured in Krippendorff’s α and majority vote balanced accuracy for each group and their combination on the test data.

Group	Number of annotators	Krippendorff’s α	Majority vote balanced accuracy
• Experienced clinicians	3	0.39	0.49
• Junior clinicians	4	0.26	0.42
Medical students	7	0.25	0.46
Combined	14	0.28	0.41

Krippendorff’s alpha for nominal data was used to quantify interrater agreement for the forced-choice facial image classifications. Raters were grouped as experienced clinicians, junior clinicians, and medical students. All raters were blinded to the clinical reference classification and were required to classify each presented photograph as positive or negative. Majority-vote classifications were generated separately for each rater group and for all raters combined. Balanced accuracy was calculated as the mean of sensitivity and specificity.

### 3.3 Stage 1 model performance

On the internal hold-out set of 51 participants, VGG16 achieved the highest balanced accuracy of 0.71 (95% CI, 0.59–0.82), with a sensitivity of 55.6% (95% CI, 39.6–70.5) and a specificity of 86.7% (95% CI, 62.1–96.3). This corresponded to 20 true-positive, 16 false-negative, 13 true-negative, and 2 false-positive classifications.

DenseNet121 showed a similar performance profile, with a balanced accuracy of 0.70 (95% CI, 0.58–0.81), sensitivity of 52.8% (95% CI, 37.0–68.0), and specificity of 86.7% (95% CI, 62.1–96.3). The model produced 19 true-positive, 17 false-negative, 13 true-negative, and 2 false-positive classifications.

MobileNetV2 achieved the highest sensitivity at 91.7% (95% CI, 78.2–97.1), but its specificity was 33.3% (95% CI, 15.2–58.3), resulting in a balanced accuracy of 0.63 (95% CI, 0.50–0.75). It produced 33 true-positive, 3 false-negative, 5 true-negative, and 10 false-positive classifications.

Thus, VGG16 and DenseNet121 were more specific but failed to identify approximately half of the participants in the low-muscle-mass class. MobileNetV2 identified most positive participants but generated substantially more false-positive classifications. The confidence intervals were wide and overlapped across models.

Model performance and individual-rater performance were summarized separately and were not subjected to a formal superiority comparison. The image-model task and the unaided human image-classification task were not equivalent, and the human experiment was not treated as validation of the models.

### 3.4 Stage 2 internal replication and validation

The independently redeveloped stage-2 pipeline was evaluated in a common 51-participant internal test partition containing 35 participants with low muscle mass and 16 with normal muscle mass. Stage 2 yielded 33 true-positive, 8 true-negative, 8 false-positive, and 2 false-negative classifications. Sensitivity was 94.3% (95% CI, 81.4%–98.4%), specificity 50.0% (95% CI, 28.0%–72.0%), overall accuracy 80.4%, positive predictive value 80.5%, negative predictive value 80.0%, and balanced accuracy 0.72 (95% bootstrap CI, 0.60–0.85).

Stage 2 achieved a balanced accuracy of similar magnitude to the strongest stage-1 model, despite independent redevelopment of the preprocessing and modelling pipeline. The operating characteristics nevertheless differed substantially: stage 2 emphasized sensitivity, whereas the strongest stage-1 model showed greater specificity. Taken together, the two stages support internal reproducibility of image-associated discrimination across independently implemented modelling approaches. A summary of the results from stage 1 and stage 2 is provided in Table 3.

**TABLE 3:** Performance of the stage-1 models and the integrated stage-2 pipeline in their respective internal evaluation sets.

Model	Metric	Class 0 (minority)	Class 1 (majority)	Macro Avg	Weighted Avg	TP	FN	TN	FP	Balanced accuracy	Sensitivity	Specificity
VGG16	Precision	0.45	0.90	0.68	0.77	20	16	13	2	71.0%	55.6%	86.7%
	Recall	0.87	0.56	0.71	0.65							
	F1-score	0.60	0.69	0.65	0.66							
MobileNetV2	Precision	0.63	0.77	0.70	0.73	33	3	5	10	63.0%	91.7%	33.3%
	Recall	0.33	0.92	0.63	0.75							
	F1-score	0.43	0.84	0.64	0.72							
DenseNet121	Precision	0.43	0.90	0.67	0.71	19	17	13	2	70.0%	52.8%	86.7%
	Recall	0.87	0.53	0.70	0.63							
	F1-score	0.58	0.67	0.62	0.64							
Stage 2 integrated region-specific pipeline	Precision	0.80	0.80	0.80	0.80	33	2	8	8	72.0%	94.3%	50.0%
	Recall	0.50	0.94	0.72	0.80							
	F1-score	0.62	0.87	0.74	0.79							

The binary target distinguished participants with normal measured muscle mass from participants with low measured muscle mass. Under the historical EWGSOP1 framework, the low-muscle-mass class comprised presarcopenia, sarcopenia, and severe sarcopenia, whereas the normal-muscle-mass class corresponded to participants classified as having no sarcopenia. Stage 1 was evaluated in a 51-participant internal hold-out set comprising 36 participants with low muscle mass and 15 with normal muscle mass. Stage 2 was evaluated in a separate 51-participant internal test set comprising 35 participants with low muscle mass and 16 with normal muscle mass. The four stage-2, convolutional neural networks were components of a single region-specific decision pipeline and were therefore not evaluated or reported here as independent standalone classifiers. Sensitivity corresponds to recall for the low-muscle-mass class and specificity to recall for the normal-muscle-mass class. Balanced accuracy was calculated as the mean of sensitivity and specificity. TP, true positive; FN, false negative; TN, true negative; FP, false positive.

### 3.5 Sensitivity analysis using strict EWGSOP1 sarcopenia

Across the complete 51-participant stage-2 test set, the historical EWGSOP1 distribution comprised 16 participants without sarcopenia, 18 with presarcopenia, 10 with sarcopenia, and 7 with severe sarcopenia. Among participants without sarcopenia, 8 of 16 were correctly classified as negative and 8 were classified as positive, corresponding to a specificity of 50.0% (95% CI, 28.0%–72.0%). The system classified 17 of 18 participants with presarcopenia as positive (94.4%; 95% CI, 74.2%–99.0%), 9 of 10 participants with sarcopenia as positive (90.0%; 95% CI, 59.6%–98.2%), and all 7 participants with severe sarcopenia as positive (100%; 95% CI, 64.6%–100%). Overall, 33 of 35 participants with low muscle mass were classified as positive.

For the stricter EWGSOP1 sensitivity analysis, participants with presarcopenia were excluded. The remaining 33 participants comprised 17 with EWGSOP1 sarcopenia or severe sarcopenia and 16 without sarcopenia. Without retraining or modification of the predefined decision rules, the locked stage-2 pipeline yielded 16 true-positive, 1 false-negative, 8 true-negative, and 8 false-positive classifications. Sensitivity was 94.1% (95% CI, 73.0%–99.0%), specificity 50.0% (95% CI, 28.0%–72.0%), and balanced accuracy 0.72 (95% bootstrap CI, 0.60–0.85). Exclusion of presarcopenia therefore did not materially change balanced discrimination, although specificity remained limited. Stage-specific classification results across the complete stage-2 internal test set, including the strict-EWGSOP1 sensitivity analysis, are summarized in Supplementary Table S8.

### 3.6 Clinical-variable comparator

In the same historical 51-participant hold-out set used for the first-generation facial models, the clinical-variable-only model yielded 29 true-positive, 7 false-negative, 13 true-negative, and 2 false-positive classifications. Sensitivity was 80.6% (95% CI, 65.0%–90.2%), specificity 86.7% (95% CI, 62.1%–96.3%), balanced accuracy 83.6% (95% bootstrap CI, 72.2%–93.1%), overall accuracy 82.4%, positive predictive value 93.5%, and negative predictive value 65.0%. AUROC was 0.91, AUPRC 0.95, and the Brier score 0.11.

The clinical-variable-only model therefore showed higher balanced accuracy than VGG16 (0.71), DenseNet121 (0.70), and MobileNetV2 (0.63) in the same hold-out participants. In the sensitivity analysis excluding reference-standard modality, the clinical comparator achieved 72.2% sensitivity, 80.0% specificity, balanced accuracy 76.1%, AUROC 0.81, and a Brier score of 0.16. Thus, substantial discriminatory information remained available from routine clinical variables even when assessment modality was excluded.

### 3.7 Post-hoc audit of the stage-2 pipeline

Grad-CAM analysis was completed for all four stage-2 components across all 51 internally evaluated participants, comprising 33 system-level true positives, 8 true negatives, 8 false positives, and 2 false negatives. Aggregate activation maps were generated separately for each outcome category.

In the full-face-no-neck component, Grad-CAM activation was not restricted to the prespecified facial-envelope proxy. Across the test set, approximately 38.9% of normalized activation was located within the facial-envelope proxy and 61.1% within the complementary background proxy. Approximately 48.1% of normalized activation occurred within the outer 10% image-border region. Because the geometric masks overlap, these percentages are descriptive and should not be summed. Background-proxy activation was particularly pronounced in true-negative cases and remained substantial in the other prediction categories.

Activation was also observed within prespecified facial subregions, including temporal, periorbital, and cheek proxy regions. Across all test cases, the corresponding mean normalized activation fractions were approximately 6.5%, 5.5%, and 9.2%, respectively. These values were interpreted descriptively because the proxy regions differ in area and were not anatomical segmentations.

Perturbation analyses further demonstrated sensitivity to both facial and non-facial image content. Background masking changed the final binary system decision in 6 of 51 cases (11.8%), outer-border masking in 3 of 51 (5.9%), temporal-proxy masking in 5 of 51 (9.8%), periorbital masking in 8 of 51 (15.7%), and cheek masking in 7 of 51 (13.7%). A 10% tighter crop changed the final system decision in 8 of 51 cases (15.7%), and grayscale conversion in 10 of 51 cases (19.6%).

### 3.8 Cohort imbalances and label-associated clinical variables

In the cleaned overall cohort, an exploratory multivariable logistic regression model including reference-standard imaging modality, body mass index, age, sex, liver cirrhosis, and inflammatory bowel disease was significantly associated with the binary low-muscle-mass classification (likelihood-ratio χ^2^ = 123.498, 7 df, p < 0.001). The model had a Nagelkerke R^2^ of 0.539, and the Hosmer–Lemeshow test did not indicate poor model fit (p = 0.410). This analysis characterized the clinical structure of the study label and did not assess the incremental predictive value of the facial image models.

Participants with low muscle mass had a lower body mass index than participants with normal muscle mass in the overall cohort (24.8 ± 3.7 vs. 28.4 ± 5.3 kg/m^2^, p < 0.001). Disease group and reference-standard imaging modality were also strongly imbalanced between the two groups (both p < 0.001). Participants assessed using magnetic resonance imaging more frequently had low muscle mass, whereas those assessed using dual-energy X-ray absorptiometry more frequently had normal muscle mass.

Similar descriptive imbalances in body mass index, disease group, and imaging modality were present in the internal hold-out set. However, inferential comparisons within this small, selected subset were not used for model development or threshold selection.

In an exploratory modality-stratified analysis of the second-generation test set, balanced accuracy was 0.63 among CT-assessed participants (n = 12; 7 positive and 5 negative), 0.48 among MRI-assessed participants (n = 29; 26 positive and 3 negative), and 0.88 among DXA-assessed participants (n = 10; 2 positive and 8 negative). Sensitivity/specificity were 85.7%/40.0% for CT, 96.2%/0.0% for MRI, and 100%/75.0% for DXA. These estimates were based on very small and highly imbalanced modality-specific cells and were interpreted descriptively rather than as reliable subgroup-validation estimates. Sex and Model for End-Stage Liver Disease score did not differ significantly between the classes. Detailed modality-stratified performance of the stage-2 pipeline is provided in Supplementary Table S7.

These associations identify disease-related, body-composition–related, and diagnostic-pathway differences that could provide alternative signals correlated with the study label. They do not demonstrate that the facial image models used these characteristics or their facial correlates for classification.

### 3.9 Exploratory laboratory analyses

Several laboratory parameters differed between the low- and normal-muscle-mass classes in unadjusted complete-case analyses. In the overall cohort, differences were observed for glucose, potassium, prothrombin time, international normalized ratio, platelet count, transferrin, interleukin-6, insulin-like growth factor 1, and gamma-glutamyl transferase. In the internal hold-out set, ferritin, fibrinogen, gamma-glutamyl transferase, and transferrin differed between the two classes.

Because laboratory-data availability varied between parameters, the analyses were not adjusted for multiple comparisons, and the laboratory variables were not linked directly to model predictions or classification errors, these findings were considered exploratory. They should not be interpreted as independent biomarkers of low muscle mass or as evidence that the facial image models used corresponding biological signals. Detailed results, including group-specific sample sizes, descriptive statistics, and unadjusted p values, are presented in Supplementary Table S4.

## 4 Discussion

This exploratory modelling study investigated whether standardized frontal clinical photographs contained information associated with low measured muscle mass in a selected clinical cohort enriched for liver cirrhosis. The analysis used a two-stage modelling framework comprising initial model development and internal hold-out evaluation followed by independent redevelopment and internal replication within the same source cohort. Unaided human visual assessment showed low interrater agreement, whereas both modelling stages demonstrated moderate image-associated discrimination. These findings support the presence of a reproducible image-associated signal within the source cohort but do not establish a sarcopenia-specific facial phenotype.

The two modelling stages addressed complementary aspects of internal model evaluation. Stage 1 assessed whether a discriminative image signal could be developed and retained in an internal hold-out set. Stage 2 tested whether similar discrimination could be recovered after independent redevelopment by a second investigator using different facial representations and partially different CNN architectures. The similar balanced accuracy across stages supports internal computational reproducibility across independently implemented pipelines.

This internal replication should not be equated with external validation. Both stages used the same clinical source population and were therefore exposed to the same cohort composition, disease associations, reference-standard pathways, and potential confounders. Furthermore, the different sensitivity-specificity profiles indicate that reproducibility of overall discrimination did not imply reproduction of the same decision function.

The human-rater experiment provides complementary descriptive context but should not be interpreted as validation of the image models. Overall agreement among the 14 raters was low, and individual-rater balanced accuracy was centered near chance performance. However, raters received no clinical information and underwent no formal training or calibration. The findings therefore demonstrate limited reproducibility of unaided image-only classification under the specific experimental conditions rather than inferiority of clinical judgment to the neural networks. The first-generation architectures showed distinct sensitivity-specificity trade-offs, but their differences should be interpreted descriptively given the small hold-out set and wide confidence intervals.

The small hold-out set resulted in broad confidence intervals. Subsequent studies should prespecify model-selection procedures and decision thresholds, assess calibration, retain participant-level data separation, and evaluate locked models in independent populations (Collins et al., 2024; Moons et al., 2025; Wiens et al., 2019).

Recent reporting frameworks further clarify the evidentiary gap between retrospective proof-of-concept model evaluation and clinically deployable diagnostic systems. STARD-AI emphasizes transparent reporting of dataset construction, the AI index test, reference-standard procedures, model evaluation, and potential sources of bias and limited generalizability (Sounderajah et al., 2025). DECIDE-AI further emphasizes that early clinical evaluation should assess actual clinical performance, safety, and human factors rather than relying on preclinical or *in silico* discrimination alone (Vasey et al., 2022). The present study remains at an earlier retrospective development and internal-evaluation stage and therefore does not provide prospective evidence for performance of the complete system in clinical use.

Recent sarcopenia-AI literature provides additional context. A 2025 systematic review by Zhang et al. identified substantial methodological heterogeneity and limited external validation among sarcopenia prediction models, underscoring that internal discrimination alone is insufficient evidence of transportability (Zhang et al., 2025). Adjacent image-based work such as Face2Bone demonstrates the technical feasibility of extracting clinically associated information from facial photographs but addresses osteoporosis rather than sarcopenia and therefore does not validate the present target or approach (Liang et al., 2025). Conversely, the recently reported SAID DSS illustrates a multimodal strategy combining several clinically relevant inputs rather than relying on a single image source (Brockhattingen et al., 2026). Together, these studies reinforce the importance of explicit outcome definition, comparison with routine clinical information, transparent performance and calibration assessment, and independent validation before clinical implementation.

The findings establish image-associated discrimination under standardized acquisition conditions but do not establish a clinically useful screening operating point. Future development should focus on external validation, consistent reference standards, calibration, and incremental value beyond routine clinical information.

Biological plausibility nevertheless remains. Sarcopenia is a clinically important complication of cirrhosis and is associated with adverse outcomes (Lai et al., 2021; Tantai et al., 2022). Muscle loss, altered adiposity, malnutrition, and general wasting may also affect craniofacial soft tissues. Previous studies have associated temporal or periorbital morphology and temporalis muscle thickness with muscle strength, body composition, or malnutrition-related phenotypes (Chen et al., 2023; Steindl et al., 2020; Wang et al., 2024). These studies support the possibility of a facial correlate but do not establish specificity for sarcopenia. In particular, the available facial studies used selected clinical populations, specialized three-dimensional imaging, or outcomes related to malnutrition rather than the low-versus-normal-muscle-mass target used here. They should therefore be interpreted as evidence of biological plausibility, not as validation of two-dimensional facial classification of low muscle mass.

Participants in the low-muscle-mass class had a lower body mass index, and disease group and reference-standard imaging modality were strongly imbalanced between classifications. Previous three-dimensional facial studies indicate that facial soft-tissue morphology may covary with muscle- and fat-loss phenotypes (Chen et al., 2023; Wang et al., 2024). However, the present study did not determine whether body mass index, underlying disease, liver disease severity, systemic inflammation, or other clinical characteristics produced visual features used by the models. These variables should therefore be interpreted as cohort and label imbalances and as potential sources of confounding, not as established image features. The models may have captured facial morphology associated with low muscle mass, but they may also have learned disease-, body-composition-, or care-pathway-related proxy signals. The exploratory clinical regression characterized the structure of the reference classification; it did not demonstrate that the image models provided incremental information beyond readily available clinical variables.

The available sample size did not permit a stable multivariable analysis establishing an independent association between the historical facial score and all candidate confounders. The present analyses therefore demonstrate the magnitude of potential confounding rather than statistically removing it. This distinction further limits any claim of target-specific facial information.

BMI and disease category were also associated with the target. Consequently, reproducible discrimination across the two modelling stages does not establish that the underlying image signal was sarcopenia-specific. Both may have exploited facial or contextual correlates of the same underlying clinical structure.

Under the original 2010 European Working Group framework, presarcopenia was defined by low muscle mass alone, sarcopenia by low muscle mass together with reduced muscle strength or impaired physical performance, and severe sarcopenia by the presence of all three components (Cruz-Jentoft et al., 2010). In the present binary analysis, these three stages were combined into one positive class. Because all three stages require low muscle mass and the negative class had normal muscle mass, the machine-learning outcome was equivalent to low versus normal measured muscle mass. The primary models should therefore be interpreted as low-muscle-mass classifiers rather than classifiers of strict EWGSOP1 or contemporary sarcopenia.

EWGSOP1 was retained as reference framework because it was prospectively specified in the parent study and formed the target definition for both modelling stages. The protocol explicitly selected the 2010 framework considering previous observations that the 2019 EWGSOP2 criteria identify a materially different subset of patients with cirrhosis (Traub et al., 2020). Retrospectively redefining the target according to EWGSOP2 would therefore constitute a separate diagnostic research question rather than a simple technical relabelling of the present models. Comparison of alternative sarcopenia definitions is addressed separately, while the present study preserves the historical EWGSOP1 target for methodological consistency (Traub et al., 2020).

Model interpretability remained limited despite the additional audit. Grad-CAM of the stage-2 pipeline identified activation within plausible facial regions, including temporal, periorbital, and cheek proxies, but substantial activation was also located outside the geometric facial envelope and near image borders. Controlled perturbation of both facial and non-facial regions altered model scores and changed the final system output in a minority of cases.

These findings argue against interpreting the classifier as a direct detector of sarcopenia-related facial anatomy. Peripheral image structure, crop geometry, background, acquisition characteristics, or other correlated contextual features may have contributed to the learned signal. Conversely, the effects of facial-region masking indicate that facial content also contributed. Neither Grad-CAM nor perturbation analysis can distinguish a muscle-specific signal from correlated effects of age, BMI, general wasting, nutritional status, disease severity, or other phenotypic features.

The technical architectures of VGG16, MobileNetV2, and DenseNet121 are described in their respective source publications (Simonyan and Zisserman, 2014; Sandler et al., 2018; Huang et al., 2017), while ImageNet documents the nonmedical image-classification context from which the pretrained weights originated (Russakovsky et al., 2015). RetinaFace adds a further model-based processing stage because its detection output determines the facial region supplied to the classifiers (Deng et al., 2020). However, these sources describe the technical provenance of the pipeline and do not identify which facial structures, acquisition characteristics, or other visual features drove predictions in the models trained for the present study. No claim can therefore be made that the classifiers relied on clinically meaningful muscle-related facial features.

Grad-CAM was used here as an exploratory model-auditing method rather than as a causal explanation of model behaviour (Selvaraju et al., 2020; AWS, 2026). The systematic analysis of true-positive, true-negative, false-positive, and false-negative cases, aggregate activation maps, and controlled perturbations demonstrated that visually plausible facial activation coexisted with substantial background and crop-edge contributions. The perturbation audit assessed sensitivity to crop geometry, peripheral image content, approximate hair- and clothing-related regions, background, image borders, and color information. Device-specific robustness could not be assessed because the present photographs were acquired under standardized clinical conditions rather than across a deliberately heterogeneous device cohort. Likewise, the dataset did not provide controlled real-world variation in illumination. Synthetic perturbations can characterize model sensitivity but cannot substitute for prospective validation across camera hardware, device-specific image processing, and real-world lighting conditions.

The present study establishes feasibility under standardized clinical acquisition conditions but does not demonstrate robustness to self-acquired smartphone photographs. Translation to such images requires prospective evaluation across camera hardware, proprietary image processing, camera-to-face distance, pose, illumination, compression, background, glasses, facial hair, and user technique (Zoltie et al., 2022; Hunt et al., 2021). Guided acquisition and automated image-quality control may reduce some of this variability but would themselves require prospective validation. Real-world evaluation across devices, users, and acquisition conditions is therefore required before a smartphone-based screening application can be considered.

Facial image analysis should not replace established case-finding or diagnostic procedures based on muscle strength, physical performance, and body composition (Cruz-Jentoft et al., 2010; Cruz-Jentoft et al., 2019; Lai et al., 2021; Giovannini et al., 2021). If externally validated and shown to provide incremental value beyond routine clinical information, it could subsequently be evaluated as a complementary pre-screening component within a multistep diagnostic pathway.

A combined clinical-plus-facial model was not fitted because unbiased development-set facial scores were not available without retraining the image models within an out-of-fold framework. Such retraining would constitute an additional *post hoc* model-development step. The independent incremental contribution of facial information therefore remains unestablished.

The use of identifiable facial images to infer an individual health state also requires safeguards beyond predictive accuracy. Relevant ethical principles include beneficence, non-maleficence, autonomy, justice, and explicability (Floridi et al., 2018). Medical-imaging ethics literature further emphasizes respect for data subjects, privacy, appropriate authorization of primary and secondary data use, task-specific development, transparent evaluation, fairness, and the allocation of responsibility between developers, clinicians, and deploying institutions (Herington et al., 2023). Current health-AI guidance additionally emphasizes fairness, universality, traceability, usability, robustness, explainability, and continuous lifecycle evaluation (Lekadir et al., 2025). For a facial application, these requirements include purpose-specific consent, minimization of image retention and secondary use, documentation of the complete processing pipeline, transparent communication of uncertainty, subgroup evaluation, and clear responsibility for confirmation and follow-up (Zoltie et al., 2022; Herington et al., 2023; Lekadir et al., 2025). Privacy-preserving or on-device processing may reduce transfer and storage risks, but it would not resolve invalid inference, proxy learning, bias, false reassurance, unnecessary referral, or unequal access to confirmatory care.

Strengths of the study include standardized image acquisition, prospectively collected clinical phenotyping, participant-level separation of development and evaluation data, comparison with human image assessment, and a two-stage modelling framework incorporating independent redevelopment for internal computational replication and structured model auditing.

Several limitations constrain interpretation. First, both modelling stages used the same underlying single-center source cohort. Stage 2 provided independent computational redevelopment and internal replication, but not validation in an external clinical population. Second, the binary target was equivalent to low versus normal measured muscle mass and should not be interpreted as strict or contemporary sarcopenia. Third, reference-standard modality, BMI, and disease composition were associated with the target and represent plausible sources of confounding. Fourth, the Grad-CAM and perturbation audit of the second-generation system demonstrated contributions from both facial and non-facial image regions, precluding a claim of anatomical or sarcopenia-specific representation. Finally, the internally evaluated samples were small, and the study did not establish external generalizability, calibration in the intended-use population, incremental benefit over clinical predictors, demographic fairness, prospective clinical utility, or robustness to self-acquired smartphone photographs.

If confirmed in larger and externally validated cohorts, facial image analysis could provide a rapid, noninvasive, and readily repeatable method of initial case finding. Rather than replacing established assessments of muscle strength, physical performance, and body composition, such an approach could help identify individuals who should undergo targeted conventional evaluation. This potential may be particularly relevant in chronic liver disease, where repeated assessment is clinically important but may be constrained by patient frailty, limited time, or dependence on imaging and trained personnel. In patients with cirrhosis being evaluated for liver transplantation, earlier recognition of low muscle mass could create an opportunity for timely conventional assessment and, where clinically indicated, nutritional support, exercise-based intervention, or prehabilitation before transplantation or other major procedures (Cruz-Jentoft et al., 2010; European Association for the Study of the Liver, 2024). A low-burden screening approach could therefore become clinically valuable if it reliably identifies at-risk patients early enough to alter their subsequent assessment and management.

Standardized facial photographs contained an algorithmically detectable signal associated with low measured muscle mass in this chronic liver disease-enriched cohort. The two-stage modelling framework yielded moderate balanced discrimination in both the initial development/hold-out stage and the independently redeveloped internal replication stage, supporting internal computational reproducibility across independently implemented pipelines within the same source cohort. However, an exploratory clinical-variable-only comparator outperformed the stage-1 facial models, and the present study therefore does not establish incremental predictive value of facial information beyond routinely available clinical variables. Clinical and diagnostic-pathway confounding and the stage-2 interpretability audit further preclude interpretation of the observed signal as anatomically specific, sarcopenia-specific, externally validated, or clinically validated. Independent external validation using a locked end-to-end pipeline and a prospectively defined clinical target is required before clinical application can be considered.

## Data Availability

The raw data supporting the conclusions of this article will be made available by the authors, without undue reservation.

## References

[B1] AWS (2026). Amazon Sagemaker Documentation. Available online at: https://docs.aws.amazon.com/sagemaker/ (Accessed 3 May 2026).

[B2] Brockhattingen K. K. Karlsson E. H. Bielefeldt T. B. R. Naemi A. Andersen-Ranberg K. Moradbeiki P. (2026). The sarcopenia artificial intelligence diagnostic decision support system (SAID DSS) – a multimodal deep learning model. BMC Geriatr. 26, 415. 10.1186/s12877-026-07005-9 41588346 PMC13032624

[B3] Chen M. Wang X. Han M. Li Y. Yu N. Long X. (2023). Temporal and periorbital depressions identified by 3D images are correlated with malnutrition phenotypes in cancer patients: a pilot study. Front. Nutr. 10, 1115079. 10.3389/fnut.2023.1115079 36992909 PMC10042485

[B4] Collins G. S. Moons K. G. M. Dhiman P. Riley R. D. Beam A. L. van Calster B. (2024). TRIPOD+AI statement: updated guidance for reporting clinical prediction models that use regression or machine learning methods. BMJ 385, e078378. 10.1136/bmj-2023-078378 38626948 PMC11019967

[B5] Cruz-Jentoft A. J. Baeyens J. P. Bauer J. M. Boirie Y. Cederholm T. Landi F. (2010). Sarcopenia: european consensus on definition and diagnosis: report of the european working group on sarcopenia in older people. Age Ageing 39, 412–423. 10.1093/ageing/afq034 20392703 PMC2886201

[B6] Cruz-Jentoft A. J. Bahat G. Bauer J. Boirie Y. Bruyère O. Cederholm T. (2019). Sarcopenia: revised European consensus on definition and diagnosis. Age Ageing 48, 16–31. 10.1093/ageing/afy169 30312372 PMC6322506

[B7] Deng J. Guo J. Ververas E. Kotsia I. Zafeiriou S. (2020). “RetinaFace: single-shot multi-level face localisation in the wild,” in 2020 IEEE/CVF Conference on Computer Vision and Pattern Recognition (CVPR) (Seattle, WA, USA: IEEE), 5202–5211. 10.1109/CVPR42600.2020.00525

[B8] European Association for the Study of the Liver (2024). EASL clinical practice guidelines on liver transplantation. J. Hepatol. 81, 1040–1086. 10.1016/j.jhep.2024.07.032 39487043

[B9] Floridi L. Cowls J. Beltrametti M. Chatila R. Chazerand P. Dignum V. (2018). AI4People-An ethical framework for a good AI society: opportunities, risks, principles, and recommendations. Minds Mach. (Dordr) 28, 689–707. 10.1007/s11023-018-9482-5 30930541 PMC6404626

[B10] Giovannini S. Brau F. Forino R. Berti A. D'Ignazio F. Loreti C. (2021). Sarcopenia: diagnosis and management, state of the art and contribution of ultrasound. J. Clin. Med. 10, 5552. 10.3390/jcm10235552 34884255 PMC8658070

[B11] Herington J. McCradden M. D. Creel K. Boellaard R. Jones E. C. Jha A. K. (2023). Ethical considerations for artificial intelligence in medical imaging: deployment and governance. J. Nucl. Med. 64, 1509–1515. 10.2967/jnumed.123.266110 37620051 PMC12782051

[B12] Huang G. Liu Z. van der Maaten L. Weinberger K. Q. (2017). “Densely connected convolutional networks,” in 2017 IEEE Conference on Computer Vision and Pattern Recognition (CVPR) (Honolulu, HI: IEEE), 21.07, 2261–2269. 10.1109/CVPR.2017.243

[B13] Hunt B. Ruiz A. Pogue B. (2021). Smartphone-based imaging systems for medical applications: a critical review. J. Biomed. Opt. 26, 040902. 10.1117/1.JBO.26.4.040902 33860648 PMC8047775

[B14] Lai J. C. Tandon P. Bernal W. Tapper E. B. Ekong U. Dasarathy S. (2021). Malnutrition, frailty, and sarcopenia in patients with cirrhosis: 2021 practice guidance by the American association for the study of liver diseases. Hepatology 74, 1611–1644. 10.1002/hep.32049 34233031 PMC9134787

[B15] Lekadir K. Frangi A. F. Porras A. R. Glocker B. Cintas C. Langlotz C. P. (2025). FUTURE-AI: international consensus guideline for trustworthy and deployable artificial intelligence in healthcare. BMJ 388, e081554. 10.1136/bmj-2024-081554 39909534 PMC11795397

[B16] Liang Q. Zhao J. Yang F. Chen X. Song Y. Shi Z. (2025). Face2Bone explainable AI model predicts osteoporosis risk from facial images in proof of concept study. Sci. Rep. 15, 40913. 10.1038/s41598-025-20462-3 41266389 PMC12635390

[B17] Marzi G. Balzano M. Marchiori D. (2024). K-Alpha calculator-krippendorff's alpha calculator: a user-friendly tool for computing Krippendorff's alpha inter-rater reliability coefficient. MethodsX 12, 102545. 10.1016/j.mex.2023.102545 39669968 PMC11636850

[B18] Montano-Loza A. J. Meza-Junco J. Prado C. M. M. Lieffers J. R. Baracos V. E. Bain V. G. (2012). Muscle wasting is associated with mortality in patients with cirrhosis. Clin. Gastroenterol. Hepatol. 10, 166–173. 10.1016/j.cgh.2011.08.028 21893129

[B19] Moons K. G. M. Damen J. A. A. Kaul T. Hooft L. Andaur Navarro C. Dhiman P. (2025). PROBAST+AI: an updated quality, risk of bias, and applicability assessment tool for prediction models using regression or artificial intelligence methods. BMJ 388, e082505. 10.1136/bmj-2024-082505 40127903 PMC11931409

[B20] Qiang J. Wu D. Du H. Zhu H. Chen S. Pan H. (2022). Review on facial-recognition-based applications in disease diagnosis. Bioeng. (Basel) 9, 273. 10.3390/bioengineering9070273 35877324 PMC9311612

[B21] Russakovsky O. Deng J. Su H. Krause J. Satheesh S. Ma S. (2015). ImageNet large scale visual recognition challenge. Int. J. Comput. Vis. 115, 211–252. 10.1007/s11263-015-0816-y

[B22] Sandler M. Howard A. Zhu M. Zhmoginov A. Chen L.-C. (2018). “MobileNetV2: inverted residuals and linear bottlenecks,” in 2018 IEEE/CVF Conference on Computer Vision and Pattern Recognition (CVPR); 18.06.2018 - 23.06.2018 (Salt Lake City, UT: IEEE), 4510–4520. 10.1109/CVPR.2018.00474

[B23] Sayer A. A. Cruz-Jentoft A. (2022). Sarcopenia definition, diagnosis and treatment: consensus is growing. Age Ageing 51, afac220. 10.1093/ageing/afac220 36273495 PMC9588427

[B24] Selvaraju R. R. Cogswell M. Das A. Vedantam R. Parikh D. Batra D. (2020). Grad-CAM: visual explanations from deep networks *via* gradient-based localization. Int. J. Comput. Vis. 128, 336–359. 10.1007/s11263-019-01228-7

[B25] Simonyan K. Zisserman A. (2014). Very Deep Convolutional Networks for Large-Scale Image Recognition. arXiv.

[B26] Sounderajah V. Guni A. Liu X. Collins G. S. Karthikesalingam A. Markar S. R. (2025). The STARD-AI reporting guideline for diagnostic accuracy studies using artificial intelligence. Nat. Med. 31, 3283–3289. 10.1038/s41591-025-03953-8 40954311

[B27] Steindl A. Leitner J. Schwarz M. Nenning K.-H. Asenbaum U. Mayer S. (2020). Sarcopenia in neurological patients: standard values for temporal muscle thickness and muscle strength evaluation. J. Clin. Med. 9, 1272. 10.3390/jcm9051272 32354003 PMC7288067

[B28] Tantai X. Liu Y. Yeo Y. H. Praktiknjo M. Mauro E. Hamaguchi Y. (2022). Effect of sarcopenia on survival in patients with cirrhosis: a meta-analysis. J. Hepatol. 76, 588–599. 10.1016/j.jhep.2021.11.006 34785325

[B29] Traub J. Bergheim I. Eibisberger M. Stadlbauer V. (2020). Sarcopenia and liver cirrhosis-comparison of the european working group on sarcopenia criteria 2010 and 2019. Nutrients 12, 547. 10.3390/nu12020547 32093198 PMC7071440

[B30] Vasey B. Nagendran M. Campbell B. Clifton D. A. Collins G. S. Denaxas S. (2022). Reporting guideline for the early-stage clinical evaluation of decision support systems driven by artificial intelligence: DECIDE-AI. Nat. Med. 28, 924–933. 10.1038/s41591-022-01772-9 35585198

[B31] Wang X. Liu Y. Rong Z. Wang W. Han M. Chen M. (2024). Development and evaluation of a deep learning framework for the diagnosis of malnutrition using a 3D facial points cloud: a cross-sectional study. JPEN. J. Parenter. Enter. Nutr. 48, 554–561. 10.1002/jpen.2643 38796717

[B32] Wiens J. Saria S. Sendak M. Ghassemi M. Liu V. X. Doshi-Velez F. (2019). Do no harm: a roadmap for responsible machine learning for health care. Nat. Med. 25, 1337–1340. 10.1038/s41591-019-0548-6 31427808

[B33] Wu D. Qiang J. Hong W. Du H. Yang H. Zhu H. (2024). Artificial intelligence facial recognition system for diagnosis of endocrine and metabolic syndromes based on a facial image database. Diabetes Metab. Syndr. 18, 103003. 10.1016/j.dsx.2024.103003 38615568

[B34] Zhang X. Lian R. Tang H. Luo S. Chen X. Lu J. (2025). Diagnostic prediction models for sarcopenia: a systematic review and meta-analysis. J. Am. Med. Dir. Assoc. 26, 105698. 10.1016/j.jamda.2025.105698 40451253

[B35] Zoltie T. Blome-Eberwein S. Forbes S. Theaker M. Hussain W. (2022). Medical photography using Mobile devices. BMJ 378, e067663. 10.1136/bmj-2021-067663 36028231 PMC9465817

