# Supplementary material for: Deep-learning analysis suggests a facial image signal associated with low muscle mass in a clinical cohort enriched for chronic liver disease: a proof-of-concept study
Source: Front Cell Dev Biol. 2026 Oct 12;14:1964880. doi: 10.3389/fcell.2026.1964880 (PMC13696313; doi:10.3389/fcell.2026.1964880)
Supplement: Supplementary file 1 [file Table1.docx]

Supplementary Table S1. Dominant handgrip strength according to sex and binary low-muscle-mass classification.

| **Sex** | **Normal muscle mass, n** | **Normal muscle mass, mean ± SD** | **Normal muscle mass, median (range)** | **Low muscle mass, n** | **Low muscle mass, mean ± SD** | **Low muscle mass, median (range)** | **Total, n** | **Total, mean ± SD** | **Total, median (range)** |
| --- | --- | --- | --- | --- | --- | --- | --- | --- | --- |
| **Female** | 36 | 22.9 ± 7.2 | 23.9  (6.0-38.9) | 60 | 24.1 ± 9.5 | 23.3  (8.7-63.6) | 96 | 23.7 ± 8.7 | 23.5  (6.0-63.6) |
| **Male** | 46 | 37.3 ± 13.3 | 39.6  (14.7-80.2) | 113 | 33.7 ± 10.3 | 32.3  (1.7-63.7) | 159 | 34.8 ± 11.3 | 34.7  (1.7-80.2) |
| **Total** | 82 | 31.0 ± 13.1 | 28.3  (6.0-80.2) | 173 | 30.4 ± 11.0 | 29.3  (1.7-63.7) | 255 | 30.6 ± 11.7 | 29.3  (1.7-80.2) |

Footnote. Dominant handgrip strength is presented in kilograms as mean ± standard deviation and median (range). The binary groups correspond to the machine-learning target of normal versus low measured muscle mass. Under the historical EWGSOP1 framework, the normal-muscle-mass group corresponded to participants classified as having no sarcopenia, whereas the low-muscle-mass group comprised participants with presarcopenia, sarcopenia, or severe sarcopenia. Handgrip strength was assessed at baseline using a Jamar hydraulic hand dynamometer. Values are presented descriptively and were not used to redefine the binary low-muscle-mass target.
