# Supplementary material for: Deep-learning analysis suggests a facial image signal associated with low muscle mass in a clinical cohort enriched for chronic liver disease: a proof-of-concept study
Source: Front Cell Dev Biol. 2026 Oct 12;14:1964880. doi: 10.3389/fcell.2026.1964880 (PMC13696313; doi:10.3389/fcell.2026.1964880)
Supplement: Supplementary file 2 [file Table2.docx]

Supplementary Table S2. Gait speed according to sex and binary low-muscle-mass classification.

| **Sex** | **Normal muscle mass, n** | **Normal muscle mass, mean ± SD** | **Normal muscle mass, median (range)** | **Low muscle mass, n** | **Low muscle mass, mean ± SD** | **Low muscle mass, median (range)** | **Total, n** | **Total, mean ± SD** |
| --- | --- | --- | --- | --- | --- | --- | --- | --- |
| **Female** | 33 | 1.2 ± 0.4 | 1.1  (0.6-2.2) | 57 | 1.1 ± 0.3 | 1.1  (0.5-1.8) | 90 | 1.1 ± 0.3 |
| **Male** | 44 | 1.1 ± 0.4 | 1.1  (0.4-2.1) | 105 | 1.0 ± 0.3 | 1.01  (0.5-2.0) | 149 | 1.1 ± 0.3 |
| **Total** | 77 | 1.1 ± 0.4 | 1.1  (0.4-2.2) | 162 | 1.1 ± 0.3 | 1.04  (0.5-2.0) | 239 | 1.1 ± 0.3 |

Footnote. Gait speed is presented in metres per second (m/s) as mean ± standard deviation and median (range). The binary groups correspond to the machine-learning target of normal versus low measured muscle mass. Under the historical EWGSOP1 framework, the normal-muscle-mass group corresponded to participants classified as having no sarcopenia, whereas the low-muscle-mass group comprised participants with presarcopenia, sarcopenia, or severe sarcopenia. Gait speed was assessed at baseline over a timed 4-m walking course. Gait-speed measurements were available for 239 of 255 participants, including 77 of 82 participants with normal muscle mass and 162 of 173 participants with low muscle mass. Values are presented descriptively and were not used to redefine the binary low-muscle-mass target.
