# Supplementary material for: Deep-learning analysis suggests a facial image signal associated with low muscle mass in a clinical cohort enriched for chronic liver disease: a proof-of-concept study
Source: Front Cell Dev Biol. 2026 Oct 12;14:1964880. doi: 10.3389/fcell.2026.1964880 (PMC13696313; doi:10.3389/fcell.2026.1964880)
Supplement: Supplementary file 3 [file Table3.docx]

Supplementary Table S3. Chair-rise test performance according to sex and binary low-muscle-mass classification.

| **Sex** | **Normal muscle mass, n** | **Normal muscle mass, mean ± SD** | **Normal muscle mass, median (range)** | **Low muscle mass, n** | **Low muscle mass, mean ± SD** | **Low muscle mass, median (range)** | **Total, n** | **Total, mean ± SD** | **Total, median (range)** |
| --- | --- | --- | --- | --- | --- | --- | --- | --- | --- |
| **Female** | 33 | 16.2 ± 8.1 | 13.2  (5.7-39.7) | 59 | 12.6 ± 5.7 | 11.8  (5.6-30.9) | 92 | 13.9± 6.8 | 12.0 (5.6-39.7) |
| **Male** | 41 | 13.9 ± 6.0 | 12.0  (6.8-31.06) | 104 | 15.5 ± 7.2 | 14.3  (5.2-53.0) | 145 | 15.0± 6.9 | 13.7 (5.2-53.0) |
| **Total** | 74 | 14.9 ± 7.1 | 12.4  (5.7  -39.7) | 163 | 14.5 ± 6.8 | 13.4  (5.2-53.0) | 237 | 14.6± 6.9 | 13.2 (5.2-53.0) |

Footnote. Chair-rise test performance is presented in seconds as mean ± standard deviation and median (range). Lower values indicate faster completion of the test. The binary groups correspond to the machine-learning target of normal versus low measured muscle mass. Under the historical EWGSOP1 framework, the normal-muscle-mass group corresponded to participants classified as having no sarcopenia, whereas the low-muscle-mass group comprised participants with presarcopenia, sarcopenia, or severe sarcopenia. Lower-extremity function was assessed at baseline using the five-times chair-rise test. Chair-rise measurements were available for 237 of 255 participants, including 74 of 82 participants with normal muscle mass and 163 of 173 participants with low muscle mass. Values are presented descriptively and were not used to redefine the binary low-muscle-mass target.
