# Supplementary material for: Deep-learning analysis suggests a facial image signal associated with low muscle mass in a clinical cohort enriched for chronic liver disease: a proof-of-concept study
Source: Front Cell Dev Biol. 2026 Oct 12;14:1964880. doi: 10.3389/fcell.2026.1964880 (PMC13696313; doi:10.3389/fcell.2026.1964880)
Supplement: Supplementary file 4 [file Table4.docx]

Supplementary Table S4. Laboratory parameters according to binary low-muscle-mass classification in the overall cohort and stage-1 internal hold-out set.

| Laboratory parameter | Overall cohort: Normal muscle mass | Overall cohort: Low muscle mass | p value | Test set: Normal muscle mass | Test set: Low muscle mass | p value |
| --- | --- | --- | --- | --- | --- | --- |
| ALB (g/dL) | n=82; 4.1 ± 0.5 | n=171; 4.2 ± 0.6 | .065 | n=15; 4.0 ± 0.7 | n=35; 4.2 ± 0.6 | .497 |
| ALT (U/L) | n=82; 33.3 ± 22.5 | n=171; 37.4 ± 31.7 | .828 | n=15; 36.1 ± 16.2 | n=35; 36.8 ± 23.0 | .743 |
| AP (U/L) | n=82; 116.0 ± 61.6 | n=171; 126.4 ± 126.1 | .218 | n=15; 118.1 ± 53.9 | n=35; 104.7 ± 48.3 | .351 |
| APTT (s) | n=81; 34.7 ± 6.3 | n=162; 33.7 ± 6.4 | .201 | n=15; 35.1 ± 6.1 | n=32; 32.8 ± 5.2 | .249 |
| AST (U/L) | n=82; 41.0 ± 25.7 | n=171; 43.1 ± 30.9 | .945 | n=15; 44.2 ± 24.6 | n=35; 42.1 ± 20.0 | .949 |
| BILIDI (mg/dL) | n=81; 0.6 ± 1.0 | n=156; 0.8 ± 2.3 | .912 | n=15; 1.0 ± 2.1 | n=26; 0.7 ± 1.0 | .542 |
| BILIG (mg/dL) | n=82; 1.1 ± 1.2 | n=170; 1.2 ± 2.7 | .605 | n=15; 1.5 ± 2.2 | n=35; 1.0 ± 1.1 | .368 |
| CA (mmol/L) | n=82; 2.4 ± 0.1 | n=170; 2.4 ± 0.2 | .702 | n=15; 2.4 ± 0.2 | n=35; 2.3 ± 0.1 | .153 |
| CHE (U/L) | n=82; 6115.5 ± 2435.1 | n=170; 6210.5 ± 2446.9 | .677 | n=15; 6010.7 ± 2349.5 | n=35; 6168.9 ± 2385.9 | .899 |
| CHOL (mg/dL) | n=82; 170.7 ± 40.6 | n=168; 168.6 ± 43.9 | .506 | n=15; 167.4 ± 38.5 | n=34; 160.1 ± 35.6 | .588 |
| CL (mmol/L) | n=82; 102.6 ± 2.9 | n=171; 102.1 ± 3.7 | .394 | n=15; 102.2 ± 3.2 | n=35; 102.9 ± 3.8 | .678 |
| CRP (mg/L) | n=82; 5.1 ± 7.6 | n=170; 6.9 ± 11.4 | .529 | n=15; 7.3 ± 8.5 | n=35; 3.5 ± 3.8 | .080 |
| FERR (ng/mL) | n=63; 171.3 ± 162.8 | n=106; 146.5 ± 178.1 | .141 | n=10; 194.0 ± 105.8 | n=11; 61.7 ± 103.8 | .007 |
| FIB (mg/dL) | n=80; 308.2 ± 101.5 | n=164; 283.8 ± 108.2 | .303 | n=15; 334.1 ± 92.8 | n=33; 233.4 ± 105.0 | .003 |
| TP (g/dL) | n=82; 7.5 ± 0.5 | n=168; 7.4 ± 0.7 | .265 | n=15; 7.4 ± 0.6 | n=34; 7.58 ± 0.8 | .632 |
| GFRCKD (mL/min/1.73 m²) | n=81; 85.1 ± 22.4 | n=171; 90.2 ± 26.4 | .319 | n=15; 85.4 ± 17.5 | n=35; 92.1 ± 21.9 | .271 |
| GGT (U/L) | n=82; 139.6 ± 154.3 | n=171; 127.4 ± 209.1 | .008 | n=15; 163.6 ± 107.6 | n=35; 99.7 ± 105.7 | .015 |
| GLUC (mg/dL) | n=82; 116.2 ± 50.2 | n=170; 98.5 ± 39.6 | .007 | n=15; 107.5 ± 35.1 | n=35; 88.8 ± 36.5 | .266 |
| HB (g/dL) | n=79; 13.3 ± 1.7 | n=160; 13.6 ± 1.8 | .362 | n=14; 14.0 ± 1.4 | n=28; 14.0 ± 1.8 | .669 |
| HKT (%) | n=79; 39.4 ± 4.7 | n=160; 40.1 ± 5.2 | .312 | n=14; 40.5 ± 4.5 | n=28; 40.8 ± 5.1 | .531 |
| K (mmol/L) | n=81; 4.1 ± 0.4 | n=171; 4.0 ± 0.3 | .006 | n=15; 4.1 ± 0.4 | n=35; 4.0 ± 0.4 | .530 |
| KREA (mg/dL) | n=82; 0.9 ± 0.3 | n=171; 0.9 ± 0.4 | .384 | n=15; 0.8 ± 0.2 | n=35; 0.9 ± 0.3 | .596 |
| LEUKO (10⁹/L) | n=80; 6.9 ± 2.9 | n=172; 6.3 ± 2.3 | .193 | n=14; 7.5 ± 3.3 | n=35; 6.1 ± 2.7 | .160 |
| NA (mmol/L) | n=81; 138.4 ± 2.8 | n=170; 138.6 ± 3.2 | .527 | n=15; 137.5 ± 3.6 | n=35; 139.4 ± 2.7 | .063 |
| PZ (%) | n=81; 84.2 ± 21.7 | n=164; 91.2 ± 22.3 | .014 | n=15; 87.0 ± 17.9 | n=33; 92.0 ± 18.5 | .469 |
| PZINR | n=81; 1.2 ± 0.2 | n=165; 1.1 ± 0.3 | .008 | n=15; 1.1 ± 0.2 | n=33; 1.1 ± 0.1 | .361 |
| THROM (10⁹/L) | n=80; 177.8 ± 101.0 | n=171; 202.9 ± 99.0 | .031 | n=14; 164.1 ± 79.3 | n=34; 177.3 ± 98.2 | .794 |
| TRF (g/L) | n=61; 2.7 ± 0.4 | n=89; 2.5 ± 0.6 | .028 | n=10; 2.6 ± 0.5 | n=3; 1.9 ± 0.3 | .042 |
| TRFSAT (%) | n=61; 27.2 ± 13.9 | n=89; 29.1 ± 14.8 | .482 | n=10; 29.8 ± 13.8 | n=3; 48.3 ± 28.1 | .446 |
| TRI (mg/dL) | n=82; 115.0 ± 54.4 | n=168; 112.6 ± 62.2 | .484 | n=15; 100.5 ± 29.9 | n=34; 108.4 ± 49.5 | .862 |
| IL6 (pg/mL) | n=62; 8.2 ± 7.7 | n=85; 10.9 ± 21.2 | .009 | n=10; 12.2 ± 11.3 | n=3; 26.4 ± 12.2 | .091 |
| WFAG (%) | n=57; 150.5 ± 49.5 | n=83; 164.3 ± 68.2 | .959 | n=9; 151.9 ± 50.1 | n=3; 139.0 ± 7.2 | .853 |
| BETA-D (ng/mL) | n=14; 22.6 ± 13.9 | n=45; 22.2 ± 20.4 | .332 | n=1; 59.5 | n=0 | n/a |
| FGF23 (RU/mL) | n=20; 58.4 ± 47.7 | n=45; 62.6 ± 110.6 | .378 | n=1; 51.9 | n=0 | n/a |
| GH (ng/mL) | n=39; 6.3 ± 21.0 | n=54; 2.2 ± 3.8 | .117 | n=5; 5.2 ± 9.3 | n=1; 4.7 | n/a |
| HEPC (ng/mL) | n=12; 10.6 ± 12.4 | n=39; 12.4 ± 9.9 | .169 | n=0 | n=0 | n/a |
| MSTN (pg/mL) | n=24; 1149.7 ± 517.3 | n=16; 1205.9 ± 453.4 | .629 | n=3; 1035.6 ± 628.2 | n=0 | n/a |
| TT (ng/mL) | n=47; 2.2 ± 2.5 | n=52; 2.8 ± 2.4 | .125 | n=6; 0.5 ± 0.7 | n=1; 6.8 | n/a |
| IGF1 (ng/mL) | n=52; 82.1 ± 44.7 | n=53; 118.5 ± 82.4 | .015 | n=8; 65.5 ± 19.4 | n=1; 29.6 | n/a |
| PTH (pg/mL) | n=45; 38.5 ± 17.4 | n=53; 42.8 ± 20.0 | .418 | n=6; 39.2 ± 17.0 | n=1; 22.2 | n/a |
| SHBG (nmol/L) | n=52; 79.6 ± 39.8 | n=52; 81.9 ± 106.5 | .183 | n=8; 80.8 ± 43.5 | n=1; 106.0 | n/a |
| VITD (ng/mL) | n=53; 25.5 ± 12.6 | n=53; 23.7 ± 10.9 | .820 | n=9; 21.2 ± 7.3 | n=1; 5.2 | n/a |
| VITD-OH2 (pg/mL) | n=24; 140.5 ± 62.8 | n=47; 127.2 ± 39.6 | .381 | n=3; 108.3 ± 47.0 | n=1; 75.0 | n/a |
| MELD | n=81; 9.1 ± 3.5 | n=164; 8.8 ± 4.1 | .237 | n=15; 8.7 ± 3.5 | n=33; 8.2 ± 3.1 | .640 |

Footnote. Values are shown as n; mean ± standard deviation. Laboratory parameters are reported separately for the overall cohort and the predefined test set comparing low and normal muscle mass participants. Muscle mass status was defined using the EWGSOP1-based clinical reference standard. p values are based on unadjusted Mann-Whitney U tests comparing sarcopenic and non-sarcopenic participants and were calculated on a complete-case basis. Parameters with fewer than three valid observations in either group were not tested inferentially and are reported as n/a. All p values are exploratory and were not adjusted for multiple testing. ALB, albumin; ALT, alanine aminotransferase; AP, alkaline phosphatase; APTT, activated partial thromboplastin time; AST, aspartate aminotransferase; BILIDI, direct bilirubin; BILIG, total bilirubin; CA, calcium; CHE, cholinesterase; CHOL, cholesterol; CL, chloride; CRP, C-reactive protein; FERR, ferritin; FIB, fibrinogen; TP, total protein; GFRCKD, estimated glomerular filtration rate; GGT, gamma-glutamyl transferase; GLUC, glucose; HB, hemoglobin; HKT, hematocrit; K, potassium; KREA, creatinine; LEUKO, leukocytes; NA, sodium; PZ, prothrombin time/Quick value; PZINR, international normalized ratio; THROM, thrombocytes; TRF, transferrin; TRFSAT, transferrin saturation; TRI, triglycerides; IL6, interleukin-6; WFAG, von Willebrand factor antigen; BETA-D, β-defensin; FGF23, fibroblast growth factor 23; GH, growth hormone; HEPC, hepcidin; MSTN, myostatin; TT, total testosterone; IGF1, insulin-like growth factor 1; PTH, parathyroid hormone; SHBG, sex hormone-binding globulin; VITD, 25-hydroxyvitamin D; VITD-OH2, 1,25-dihydroxyvitamin D; MELD, Model for End-Stage Liver Disease.
