# Supplementary material for: Deep-learning analysis suggests a facial image signal associated with low muscle mass in a clinical cohort enriched for chronic liver disease: a proof-of-concept study
Source: Front Cell Dev Biol. 2026 Oct 12;14:1964880. doi: 10.3389/fcell.2026.1964880 (PMC13696313; doi:10.3389/fcell.2026.1964880)
Supplement: Supplementary file 5 [file Table5.docx]

Supplementary Table S5. Model configuration and computational characteristics of the two-stage modelling framework

A. Stage 1: initial model development and internal hold-out evaluation

| **Parameter** | **VGG16** | **MobileNetV2** | **DenseNet121** |
| --- | --- | --- | --- |
| Pretraining | ImageNet-1K | ImageNet-1K | ImageNet-1K |
| Input size | 224 × 224 RGB | 224 × 224 RGB | 224 × 224 RGB |
| Input normalization | ImageNet mean/SD | ImageNet mean/SD | ImageNet mean/SD |
| ImageNet mean | [0.485, 0.456, 0.406] | [0.485, 0.456, 0.406] | [0.485, 0.456, 0.406] |
| ImageNet SD | [0.229, 0.224, 0.225] | [0.229, 0.224, 0.225] | [0.229, 0.224, 0.225] |
| Tensor format | Channels first (NCHW) | Channels first (NCHW) | Channels first (NCHW) |
| Convolutional feature extractor | Frozen | Frozen | Frozen |
| Classification head | FC → ReLU → dropout → 1 logit | FC → ReLU → dropout → 1 logit | FC → ReLU → dropout → 1 logit |
| Fully connected units | 128 | 256 | 128 |
| Dropout | 0.30 | 0.50 | 0.50 |
| Learning rate | 1 × 10⁻⁴ | 1 × 10⁻³ | 1 × 10⁻⁴ |
| Weight decay | 0.01 | 0.001 | 0.01 |
| Optimizer | Adam | Adam | Adam |
| Loss function | Binary cross-entropy | Binary cross-entropy | Binary cross-entropy |
| Class weighting | Inverse class-frequency weighting | Inverse class-frequency weighting | Inverse class-frequency weighting |
| Hyperparameter selection | Repeated stratified 5-fold cross-validation | Repeated stratified 5-fold cross-validation | Repeated stratified 5-fold cross-validation |
| Search strategy | Grid search | Grid search | Grid search |
| Learning-rate scheduler | ReduceLROnPlateau | ReduceLROnPlateau | ReduceLROnPlateau |
| Early stopping | Yes | Yes | Yes |
| Decision threshold | 0.55 | 0.40 | 0.50 |
| Best validation balanced accuracy | 0.8159 | 0.8104 | 0.7775 |
| Development set | 204 participants | 204 participants | 204 participants |
| Development class distribution | 137 low / 67 normal muscle mass | 137 low / 67 normal muscle mass | 137 low / 67 normal muscle mass |
| Internal hold-out set | 51 participants | 51 participants | 51 participants |
| Hold-out class distribution | 36 low / 15 normal muscle mass | 36 low / 15 normal muscle mass | 36 low / 15 normal muscle mass |

B. Stage 2: independent redevelopment and internal replication

| **Parameter** | **Full-face no-neck** | **Masked full-face no-neck** | **Aligned midface** | **Aligned eye-cheek** |
| --- | --- | --- | --- | --- |
| System key | no_neck | masked | midface | eye_cheek |
| Region of interest | full_face_no_neck | full_face_no_neck_masked | aligned_midface | aligned_eye_cheek |
| Architecture | EfficientNet-B0 | EfficientNet-B0 | DenseNet121 | DenseNet121 |
| Input size | 224 × 224 RGB | 224 × 224 RGB | 224 × 224 RGB | 224 × 224 RGB |
| Normalization | ImageNet mean/SD | ImageNet mean/SD | ImageNet mean/SD | ImageNet mean/SD |
| Classification-head units | 128 | 128 | 256 | 256 |
| Dropout | 0.30 | 0.30 | 0.50 | 0.50 |
| Batch size | 8 | 8 | 4 | 4 |
| Learning rate | 1 × 10⁻⁵ | 1 × 10⁻⁵ | 8 × 10⁻⁶ | 8 × 10⁻⁶ |
| Selected epoch | 3 | 4 | 4 | 6 |
| Checkpoint selection score | 0.9309 | 0.8676 | 0.9956 | 0.9000 |
| Negative-class weight | 2.0 | 2.0 | 2.0 | 2.0 |
| Additional hard-negative weight | 2.0 | 2.0 | 2.0 | 2.0 |
| Training mode | Last-block specificity tuning | Last-block specificity tuning | Last-block specificity tuning | Last-block specificity tuning |
| Model output | One logit → sigmoid | One logit → sigmoid | One logit → sigmoid | One logit → sigmoid |

C. Retained computational environment for stage 2

| **Software / environment** | **Version** |
| --- | --- |
| Windows Subsystem for Linux | WSL2 2.3.26.0 |
| Linux distribution | Ubuntu 24.04.4 LTS |
| Linux kernel | 5.15.167.4-microsoft-standard-WSL2 |
| Python | 3.12.3 |
| PyTorch | 2.12.1 |
| torchvision | 0.27.1 |
| NumPy | 2.5.0 |
| pandas | 3.0.3 |
| Pillow | 12.2.0 |
| scikit-learn | 1.9.0 |
| OpenCV | 4.13.0.92 |
| Matplotlib | 3.11.0 |
| Execution device for Grad-CAM audit | CPU |

Footnote. Stage 1 comprised three independently evaluated ImageNet-pretrained convolutional neural-network architectures, whereas stage 2 comprised four region-specific convolutional neural-network components integrated within a single decision pipeline. Stage-1 model development and hyperparameter selection were restricted to the 204-participant development set, and final performance was evaluated in the 51-participant internal hold-out set. Stage-2 checkpoint metadata and software versions refer to the retained computational environment used for the reproducible stage-2 analysis and model audit. Composite stage-2 decision rules are reported separately in Supplementary Table S6.
