# Supplementary material for: Deep-learning analysis suggests a facial image signal associated with low muscle mass in a clinical cohort enriched for chronic liver disease: a proof-of-concept study
Source: Front Cell Dev Biol. 2026 Oct 12;14:1964880. doi: 10.3389/fcell.2026.1964880 (PMC13696313; doi:10.3389/fcell.2026.1964880)
Supplement: Supplementary file 6 [file Table6.docx]

Supplementary Table S6. Composite decision rules of the integrated stage-2 region-specific pipeline

| **System output** | **Component scores** | **Definition** | **Decision threshold / rule** | **Role in final binary decision** |
| --- | --- | --- | --- | --- |
| Sensitive score | Full-face no-neck + aligned eye-cheek | Mean of the full-face-no-neck and aligned eye-cheek component probabilities | Positive if ≥0.44 | Contributes directly to the final binary decision |
| Risk score | Full-face no-neck + aligned midface | Mean of the full-face-no-neck and aligned midface component probabilities | Positive if ≥0.54 | Contributes directly to the final binary decision |
| High-specificity score | Masked full-face no-neck + aligned eye-cheek | Mean of the masked full-face-no-neck and aligned eye-cheek component probabilities | Positive if ≥0.60 and the risk criterion is simultaneously fulfilled | Additional high-specificity output; not required for the primary binary decision |
| Final binary stage-2 decision | Sensitive criterion + risk criterion | Integrated system-level classification based on the prespecified composite rules | Positive if sensitive-positive OR risk-positive; otherwise negative | Primary binary output used for the stage-2 performance analyses |

Footnote. The stage-2 pipeline comprised four convolutional neural-network components: full-face no-neck, masked full-face no-neck, aligned midface, and aligned eye-cheek. Each component generated a probability after sigmoid transformation of the model logit. Composite scores were calculated from the arithmetic mean of the specified component probabilities. For the primary binary evaluation reported in the manuscript, a participant was classified as positive when either the sensitive criterion or the risk criterion was fulfilled. The high-specificity score represented an additional system output and was not required for classification as positive in the primary binary analysis. The decision thresholds were prespecified for the retained stage-2 pipeline and were applied without modification during the reported internal evaluation and strict-EWGSOP1 sensitivity analysis.
