# Supplementary material for: Deep-learning analysis suggests a facial image signal associated with low muscle mass in a clinical cohort enriched for chronic liver disease: a proof-of-concept study
Source: Front Cell Dev Biol. 2026 Oct 12;14:1964880. doi: 10.3389/fcell.2026.1964880 (PMC13696313; doi:10.3389/fcell.2026.1964880)
Supplement: Supplementary file 7 [file Table7.docx]

Supplementary Table S7. Modality-stratified performance of the stage-2 pipeline

| Reference-standard modality | n | Low muscle mass, n | Normal muscle mass, n | Sensitivity | Specificity | Balanced accuracy |
| --- | --- | --- | --- | --- | --- | --- |
| CT | 12 | 7 | 5 | 85.7% | 40.0% | 0.63 |
| MRI | 29 | 26 | 3 | 96.2% | 0.0% | 0.48 |
| DXA | 10 | 2 | 8 | 100.0% | 75.0% | 0.88 |

Footnote. Performance estimates are descriptive because the modality-specific subsets were small and highly imbalanced. In particular, the MRI subgroup contained only three participants with normal muscle mass, whereas the DXA subgroup contained only two participants with low muscle mass. These analyses were not used for model development, threshold selection, or subgroup-specific model optimization and should not be interpreted as evidence of modality-specific validation. CT, computed tomography; MRI, magnetic resonance imaging; DXA, dual-energy X-ray absorptiometry.
