# Supplementary material for: Deep-learning analysis suggests a facial image signal associated with low muscle mass in a clinical cohort enriched for chronic liver disease: a proof-of-concept study
Source: Front Cell Dev Biol. 2026 Oct 12;14:1964880. doi: 10.3389/fcell.2026.1964880 (PMC13696313; doi:10.3389/fcell.2026.1964880)
Supplement: Supplementary file 8 [file Table8.docx]

Supplementary Table S8. Stage-2 classification according to EWGSOP1 category

| **EWGSOP1 category** | **n** | **Predicted positive** | **Predicted negative** | **Stage-specific measure** |
| --- | --- | --- | --- | --- |
| No sarcopenia | 16 | 8 | 8 | Specificity 50.0% |
| Presarcopenia | 18 | 17 | 1 | Positive detection rate 94.4% |
| Sarcopenia | 10 | 9 | 1 | Positive detection rate 90.0% |
| Severe sarcopenia | 7 | 7 | 0 | Positive detection rate 100.0% |
| All low-muscle-mass stages | 35 | 33 | 2 | Sensitivity 94.3% |
| Strict EWGSOP1 sarcopenia* | 17 | 16 | 1 | Sensitivity 94.1% |

*Sarcopenia and severe sarcopenia combined; presarcopenia excluded.

Footnote. The historical EWGSOP1 categories are shown to characterize stage-2 performance across the complete 51-participant internal test set. The primary binary machine-learning target combined presarcopenia, sarcopenia, and severe sarcopenia as the low-muscle-mass class. Stage-specific positive detection rates are reported descriptively and should not be interpreted as balanced accuracy because individual positive EWGSOP1 categories do not contain a negative reference group. The strict-EWGSOP1 sensitivity analysis combined sarcopenia and severe sarcopenia as positive and excluded presarcopenia.
